# Transcriptomics and metabolomics of engineered *Synechococcus elongatus* during photomixotrophic growth

**DOI:** 10.1186/s12934-022-01760-1

**Published:** 2022-03-05

**Authors:** Lin-Rui Tan, Yi-Qi Cao, Jian-Wei Li, Peng-Fei Xia, Shu-Guang Wang

**Affiliations:** 1 Shandong Key Laboratory of Water Pollution Control and Resource Reuse, School of Environmental Science and Engineering, Shandong University, Qingdao, 266237 China; 2 Sino-French Research Institute for Ecology and Environment (ISFREE), Shandong University, Qingdao, 266237 China

**Keywords:** *Synechococcus elongatus*, Photomixotrophic, Transcriptomics, Metabolomics, Glucose, Xylose

## Abstract

**Background:**

Converting carbon dioxide (CO_2_) into value-added chemicals using engineered cyanobacteria is a promising strategy to tackle the global warming and energy shortage issues. However, most cyanobacteria are autotrophic and use CO_2_ as a sole carbon source, which makes it hard to compete with heterotrophic hosts in either growth or productivity. One strategy to overcome this bottleneck is to introduce sugar utilization pathways to enable photomixotrophic growth with CO_2_ and sugar (e.g., glucose and xylose). Advances in engineering mixotrophic cyanobacteria have been obtained, while a systematic interrogation of these engineered strains is missing. This work aimed to fill the gap at omics level.

**Results:**

We first constructed two engineered *Synechococcus elongatus* YQ2-gal and YQ3-xyl capable of utilizing glucose and xylose, respectively. To investigate the metabolic mechanism, transcriptomic and metabolomic analysis were then performed in the engineered photomixotrophic strains YQ2-gal and YQ3-xyl. Transcriptome and metabolome of wild-type *S. elongatus* were set as baselines. Increased abundance of metabolites in glycolysis or pentose phosphate pathway indicated that efficient sugar utilization significantly enhanced carbon flux in *S. elongatus* as expected. However, carbon flux was redirected in strain YQ2-gal as more flowed into fatty acids biosynthesis but less into amino acids. In strain YQ3-xyl, more carbon flux was directed into synthesis of sucrose, glucosamine and acetaldehyde, while less into fatty acids and amino acids. Moreover, photosynthesis and bicarbonate transport could be affected by upregulated genes, while nitrogen transport and assimilation were regulated by less transcript abundance of related genes in strain YQ3-xyl with utilization of xylose.

**Conclusions:**

Our work identified metabolic mechanism in engineered *S. elongatus* during photomixotrophic growth, where regulations of fatty acids metabolism, photosynthesis, bicarbonate transport, nitrogen assimilation and transport are dependent on different sugar utilization. Since photomixotrophic cyanobacteria is regarded as a promising cell factory for bioproduction, this comprehensive understanding of metabolic mechanism of engineered *S. elongatus* during photomixotrophic growth would shed light on the engineering of more efficient and controllable bioproduction systems based on this potential chassis.

**Supplementary Information:**

The online version contains supplementary material available at 10.1186/s12934-022-01760-1.

## Background

Cyanobacteria are gaining popularity as microbial cell factories due to the capability of directly converting carbon dioxide (CO_2_) into value-added products, providing a potential solution for reducing the emission of greenhouse gases and the reliance on fossil fuels [1, 2]. Current studies showed that a wide spectrum of chemicals, such as alcohols, acids and alkanes, have been successfully produced by engineered cyanobacteria [3]. However, most cyanobacteria (e.g., *Synechococcus elongatus*) can only use inorganic CO_2_ as carbon source [4, 5], which inevitably limits the growth and productivity of cyanobacteria and makes it challenging for industrialization.

Two attempts have been made to overcome this challenge in cyanobacteria. One is to improve CO_2_ fixation by engineering RuBisCO (ribulose-1,5-bisphosphate carboxylase/oxygenase), the key enzyme in Calvin cycle, for higher carboxylation activity [6–8]. The other is to enable the utilization of organic carbon source (e.g., glucose and xylose) via introducing sugar utilization pathways and allow photomixotrophic growth of cyanobacteria [9]. During growth with light, CO_2_ and sugar, the productivity of chemicals and growth of cyanobacteria can be considerably increased. For instance, xylose utilization significantly enhanced growth of cyanobacteria and enabled a 64% increase of ethylene productivity to 9.86 mL/L over photoautotrophic production [10]. Efficient utilization of glucose and CO_2_ could increase production of 2,3-butanediol (23BD) to as much as 12.6 g/L in cyanobacteria [11].

As a representative, the model cyanobacterium *Synechococcus elongatus* PCC7942 (hereafter abbreviated as *S. elongatus* unless otherwise specified) has been engineered to utilize organic carbon with CO_2_ and sunlight, and has confirmed better performance in growth and productivity [12, 13]. The introduction of sugar utilization pathway allows *S. elongatus* to use glucose or xylose as a supplementary carbon source, therefore increasing the carbon flux and leading to higher production of designed products (e.g., 23BD) or accumulation of biomass [14]. Moreover, increased sugar utilization was investigated to further enhance productivity [11]. Although progresses have been made in this endeavor, the impact of extra carbon on the metabolisms of *S. elongatus* at omics level is still unclear. A systematic analysis of the engineered photomixotrophic cyanobacteria at molecular levels is imperative to further explore the potentials of this synthetic strategy.

Here, we engineered two *S. elongatus* strains YQ2-gal and YQ3-xyl capable of utilizing glucose and xylose, respectively. Transcriptomic and metabolomic analysis were performed in these strains under photomixotrophic condition with corresponded sugar using wild-type *S. elongatus* (WT) under photoautotrophic condition as baselines. Differentially expressed genes (DEGs) and differential metabolites were analyzed to capture the transcriptional perturbations and metabolic changes. Specifically, regulations in carbon metabolic pathways, nitrogen assimilation, membrane transport, fatty acids and amino acids biosynthesis were elucidated. Since these metabolic pathways provides precursors or energy for chemical production in cyanobacteria, potential effect of sugar utilization on photomixotrophic bioproduction in engineered *S. elongatus* was further discussed. Our study will fill the gap between metabolic engineering and the biology of synthetic photomixotrophic *S. elongatus*, which will not only provide new insights into the physiology and genetics of cyanobacteria, but also offer guidance to the metabolic engineering of this promising microbial chassis.

## Results and discussion

### Growth profile of engineered *S. elongatus* with glucose and xylose

**Fig. 1:**
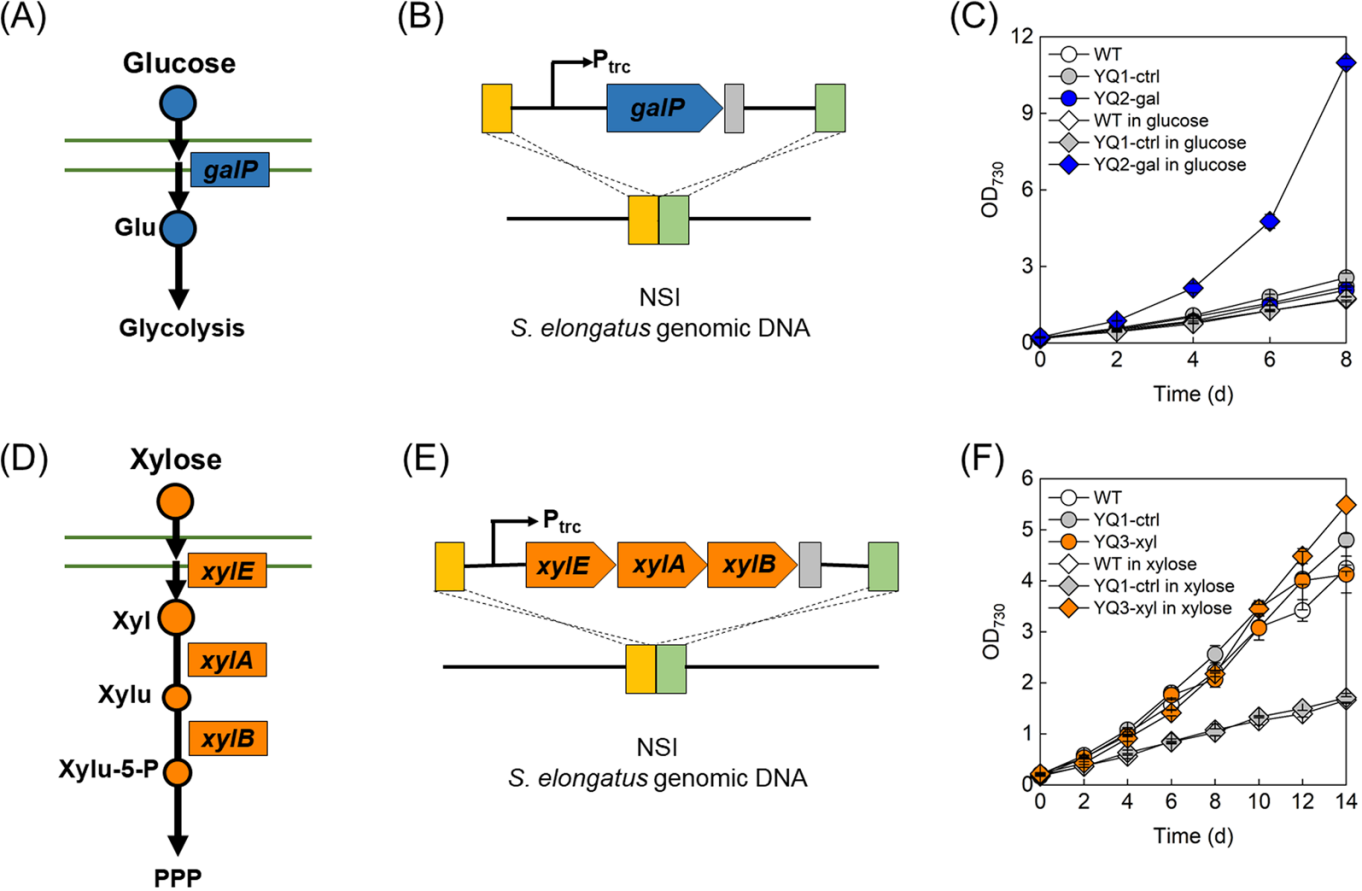
Introduction of glucose transporter or xylose degradation pathway to *S. elongatus*. Synthetic glucose transport in *S. elongatus* (**A**). Schematic representation of glucose transporter gene *galP* into *S. elongatus* genome (**B**) and growth profile of WT (empty circle), YQ1-ctrl (gray circle) and YQ2-gal (blue circle) with and without 5 g/L glucose in BG-11 medium (**C**). Synthetic xylose degradation pathway in *S. elongatus* (**D**). Schematic representation of xylose degradation pathway into *S. elongatus* genome (**E**) and growth profile of WT (empty circle), YQ1-ctrl (gray circle) and YQ3-xyl (orange circle) with and without 5 g/L xylose in BG-11 medium (**F**). Growth assays were conducted in duplicate and error bars denoted the standard deviation from the means of independent experiments. Glu, glucose; Xyl, xylose; Xylu, xylulose; Xylu-5-P, xylulose-5-phosphate; PPP, pentose phosphate pathway; NSI, neutral site I

It was reported that efficient uptake of glucose is the missing factor that hinders the utilization of glucose in *S. elongatus* (Fig. 1A) [14]. Therefore, an engineered strain harboring a glucose transporter was constructed and denoted as YQ2-gal. Specifically, *galP* gene from *Escherichia coli* was integrated into *S. elongatus* chromosome at neutral site I (NSI) under the control of *P*_*trc*_ promoter (Fig. 1B). A strain constructed via same construction process as YQ2-gal but without *galP* expression was denoted as YQ1-ctrl. Growth assays were conducted under continuous illumination of 2000–3000 lx at 30 °C and cell growth was recorded at OD_730_ every 48 h [15]. Strains WT, YQ1-ctrl and YQ2-gal showed similar growth profiles with a growth rate of 0.28/day based on OD_730_ in BG-11 medium without glucose (Fig. 1C). In the presence of 5 g/L glucose, the growth of WT and YQ1-ctrl were not affected, while growth rate showed a significant increase in YQ2-gal with efficient utilization of glucose (Fig. 1C and Additional file 1: Fig. S1A), reaching to about 5.97 times as much as WT and YQ1-ctrl.

To efficiently utilize xylose in *S. elongatus*, *xylEAB* operon from *E. coli* encoding xylose transporter, xylose isomerase and xylulokinase was integrated into *S. elongatus* chromosome at neutral site I (NSI), resulting in strain YQ3-xyl (Fig. 1D and E) [9]. Growth profiles of WT, YQ1-ctrl and YQ3-xyl were obtained under cultivation with continuous illumination of 2000–3000 lx and recorded every 48 h. Similar growth rate of 0.32/day was detected in WT, YQ1-ctrl and YQ3-xyl in BG-11 medium without xylose, while with supplementation of 5 g/L xylose, WT and YQ1-ctrl grew at a rate of 0.12/day, which is lower than that in photoautotrophic condition (Fig. 1F). Previous research has suggested that an endogenous xylose uptake system existed in wild-type cyanobacteria, while missed xylose isomerase and xylulokinase for converting xylose to central metabolites may cause metabolic imbalance [9, 10, 16], which results in a growth defect in *S. elongatus*. However, growth defect was not detected in YQ3-xyl with a complete xylose degradation pathway. This was consistent with xylose uptake profiles in Additional file 1: Fig. S1B that xylose slightly changed in WT and YQ1-ctrl, while showed significant decrease in YQ3-xyl. In the presence of xylose, YQ3-xyl even grew faster and the growth rate was 1.22 times over WT and YQ1-ctrl without xylose and 3.27 times over WT and YQ1-ctrl with the addition of xylose throughout the entire testing period of 14 days (Fig. 1F). YQ2-gal and YQ3-xyl grew well with corresponded sugar, suggesting sugar degradation pathway was successfully installed.

### Overview of transcriptomics and metabolomics during photomixotrophic growth

Synthetic *S. elongatus* with efficient sugar utilization is a promising candidate for photomixotrophic study and always compared with photoautotrophic wild type for biomass and chemical production [9, 13, 14, 17]. A better understanding of metabolic alteration of engineered photomixotrophic *S. elongatus* from WT would provide insights for a more efficient and controllable photomixotrophic system. Therefore, transcriptomic and metabolomic analysis were performed in YQ2-gal and YQ3-xyl. Transcriptome and metabolome of WT during photoautotrophic growth were set as baselines. Strains for analysis were collected at exponential phase when significant difference of growth and sugar uptake could be detected (Fig. 1 and Additional file 1: Fig. S1). Specifically, YQ2-gal was cultivated with glucose for 8 days before collection and YQ3-xyl was cultivated with xylose for 10 days before collection when YQ3-xyl significantly consumed xylose. WT was collected under the same cultivation condition but without sugar. Through the principal component analysis (PCA) of transcriptomes and metabolomes, distinct clusters of genes and metabolites were detected in YQ2-gal or YQ3-xyl and WT (Additional file 1: Figs. S2 and S3).

**Fig. 2:**
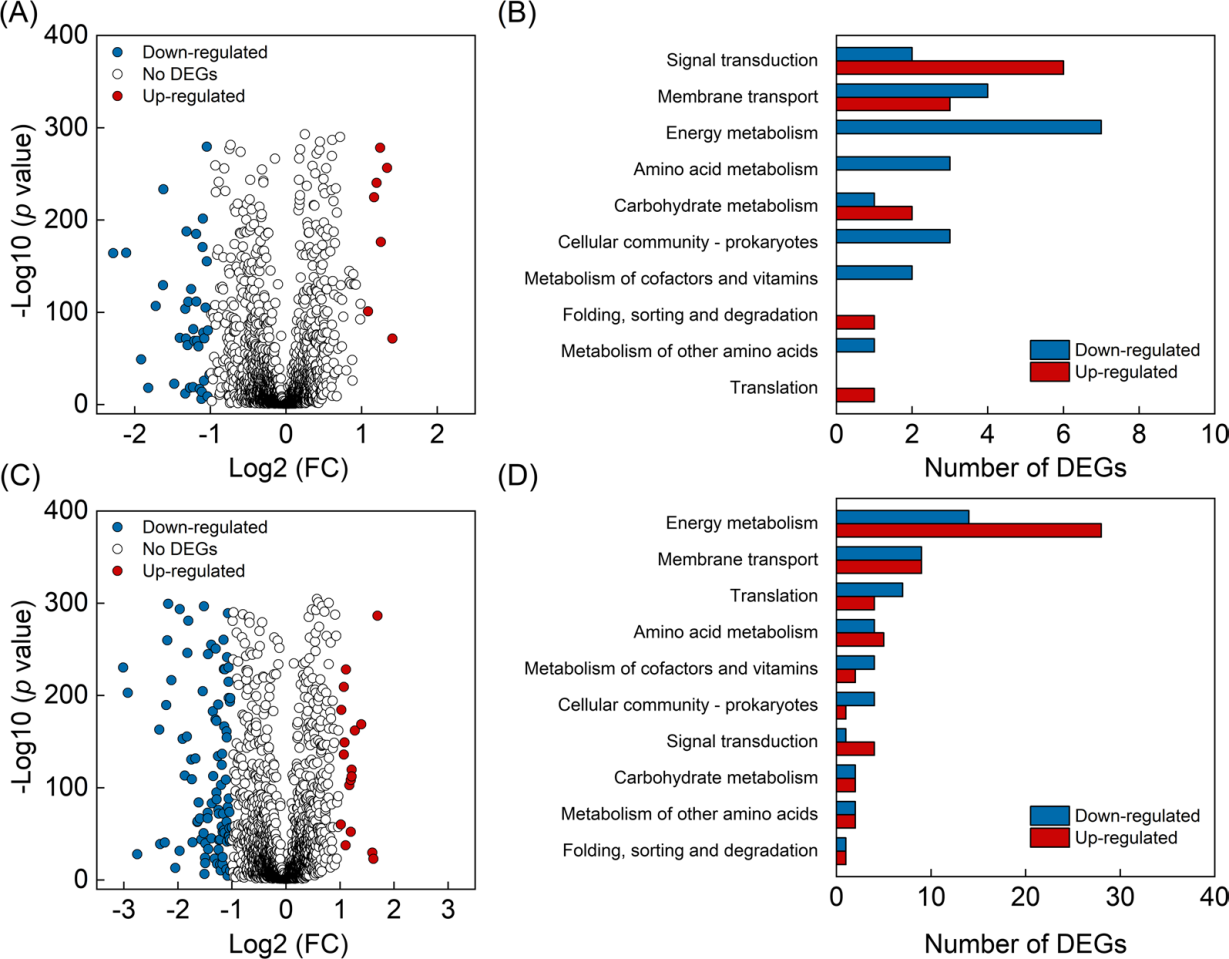
Volcano graph of DEGs and the quantitative distribution of DEGs based on annotation to KEGG database in YQ2-gal (**A**, **B**) and YQ3-xyl (**C**, **D**) compared with WT. In volcano graph (**A**, **C**), red represents upregulated genes; gray represents no significant changes; blue represents down regulated genes. FC, fold change

We found 96 DEGs with Log2 of fold change (FC) ≥ 1 or ≤ − 1 and *p* value < 0.001 in YQ2-gal (Fig. 2A), where the regulated genes were mostly annotated as hypothetical and other functional proteins with no assignment to KEGG orthology database (Additional file 2). Other DEGs were mainly related with signal transduction (8.3%) and membrane transport (7.3%) (Fig. 2B) [18]. Among 273 DEGs with Log2 (FC) ≥ 1 or ≤ − 1 and *p* value < 0.001 in YQ3-xyl (Fig. 2C), the largest categories referred to KEGG orthology database are hypothetical and other functions, energy metabolism and membrane transport, comprising 41.4%, 26.7%, 15.4% and 6.6% of the total quantity of DEGs, respectively (Fig. 2D and Additional file 3) [18].

**Fig. 3:**
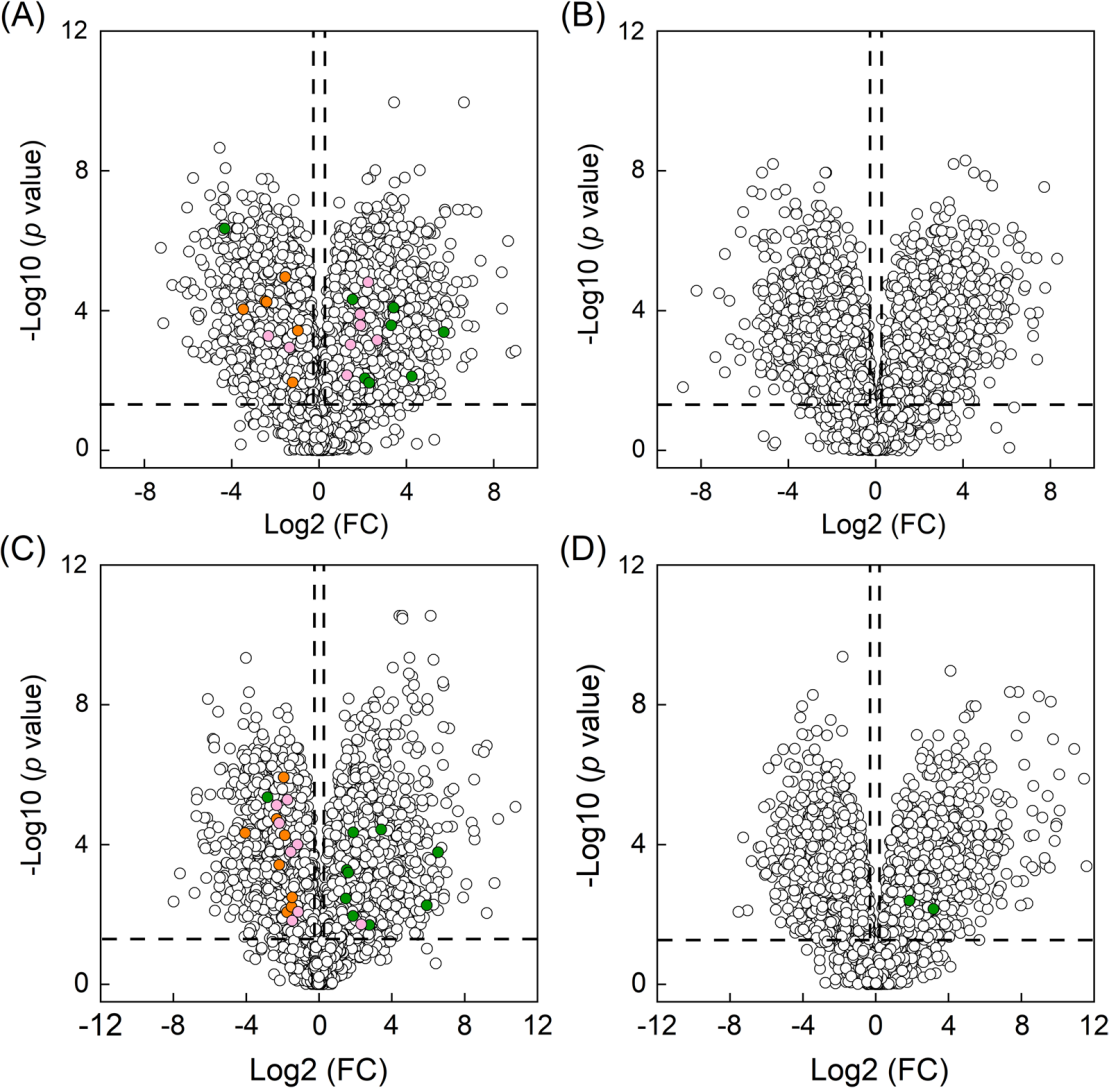
Volcano plot depicts the variations of metabolite abundance in YQ2-gal versus WT (**A**, **B**) and YQ3-xyl versus WT (**C**, **D**) according to the − log10 (*p* value). Specifically, **A** and **C** showed the results measured in positive ion mode, **B** and **D** showed the results measured in negative ion mode. Horizontal dash line represented *p* = 0.05 and vertical dash lines represented FC of 0.8333 and 1.2, respectively. Variations of metabolites with FC ≤ 0.8333 or ≥ 1.2 and *p* < 0.05 indicated as highly regulated. Green color represents highly regulated metabolites in central carbon metabolism; pink color represents highly regulated fatty acids; orange color represents highly regulated amino acids

To investigate the changes of metabolites driving by sugar utilization, differential metabolites identified with FC ≥ 1.2 or ≤ 0.833 and *p* value < 0.05 were analyzed. Since sugar was mainly metabolized via glycolysis, pentose phosphate pathway and tricarboxylic acid (TCA) cycle, we first focused on metabolites in those pathways (Fig. 3). Metabolites in Calvin cycle were then investigated as they are the major participants for CO_2_ fixation in cyanobacteria (Fig. 3). Other compounds, such as fatty acids, amino acids, sucrose and amino sugar, were also analyzed. They are produced from metabolites in carbon metabolic pathways and are important precursors for chemical production in *S. elongatus*. Variations of their abundance will help understand distribution of carbon flux and provide insights into chemical production in engineered *S. elongatus* under photomixotrophic condition (Fig. 3).

### Regulations of central metabolism in engineered *S. elongatus* during photomixotrophic growth with glucose

**Fig. 4:**
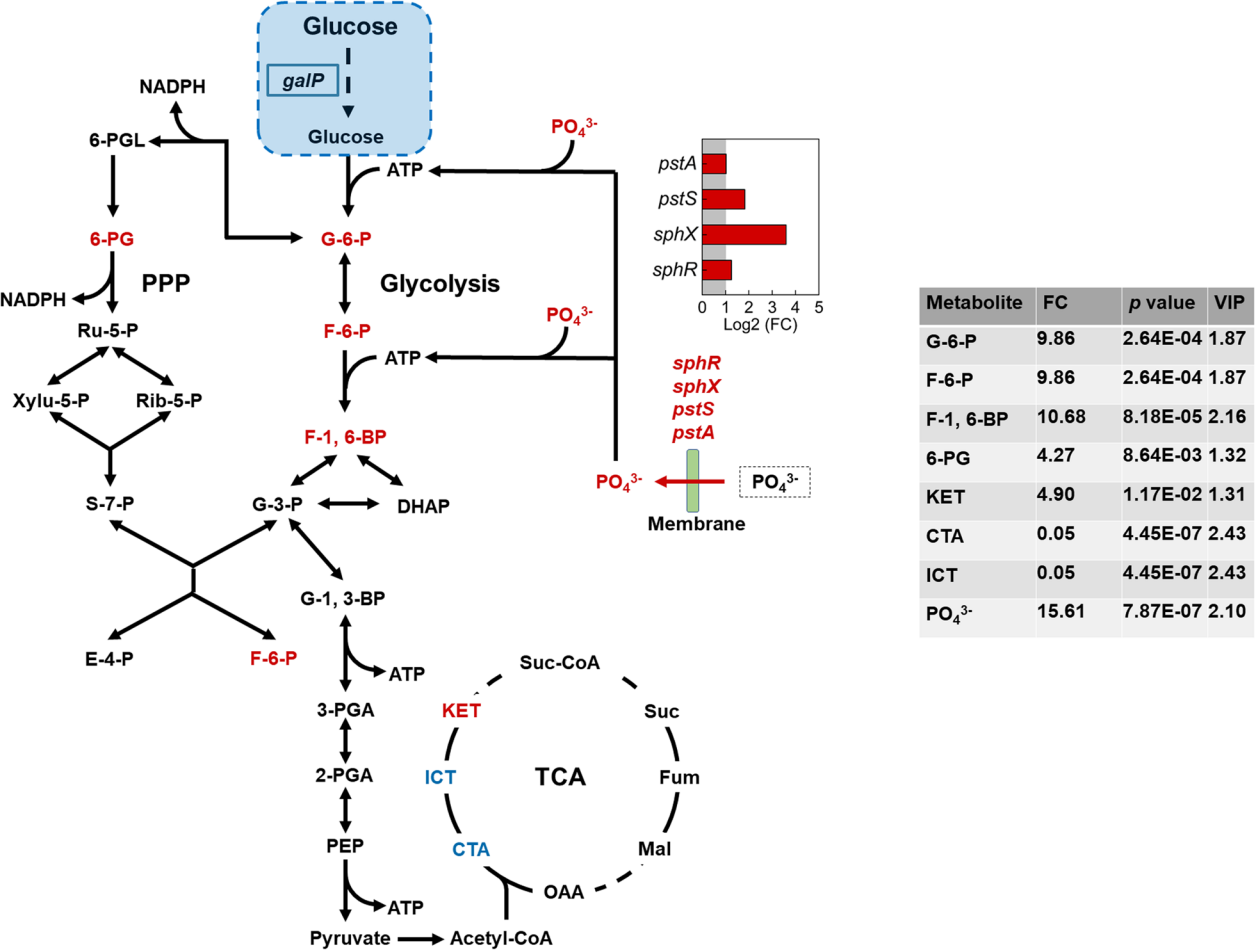
Overview of DEGs and metabolite abundance in central metabolism in YQ2-gal compared with WT. DEGs were identified with Log2 of fold change (FC) ≥ 1 or ≤ − 1 and *p* < 0.001 and metabolites driving difference were identified by reaching variable importance for the projection (VIP) ≥ 1, FC ≥ 1.2 or ≤ 0.833 and *p* < 0.05. Red color represents highly upregulated metabolites or genes and blue color represents highly downregulated metabolites or genes. G-6-P, Glucose 6-phosphate; F-6-P, Fructose 6-phosphate; F-1,6-BP, Fructose 1,6-bisphosphate; G-3-P, Glyceraldehyde 3-phosphate; DHAP, Dihydroxyacetone phosphate; G-1,3-BP, Glycerate 1,3-diphosphate; 3-PGA, 3-Phosphoglycerate; 2-PGA, 2-Phospho-(R)-glycerate; PEP, Phosphoenolpyruvate; 6-PGL, Glucono-1,5-lactone 6-phosphate; 6-PG, 6-Phospho-d-gluconate; Ru-5-P, Ribulose 5-phosphate; Rib-5-P, Ribose 5-phosphate; S-7-P, Sedoheptulose 7-phosphate; E-4-P, erythrose 4-phosphate; CTA, citrate; ICT, isocitrate; KET, 2-ketoglutarate; PPP, pentose phosphate pathway; tricarboxylic acid cycle, TCA cycle

Glycolysis is a primary pathway for glucose metabolism in microorganisms and could be affected in YQ2-gal while utilizing glucose [19, 20]. Genes in glycolysis were not differentially expressed in YQ2-gal compared with WT, while increased abundances of phosphorylated metabolites in glycolysis, such as glucose 6-phosphate (G-6-P), fructose 6-phosphate (F-6-P) and fructose 1,6-bisphosphate (F-1, 6-BP), were detected (Fig. 4). Phosphate could affect phosphorylation in glycolysis by generating ATP, thus providing energy [21, 22]. In YQ2-gal, increased phosphate with FC of 15.61 with *p* value < 0.05 was detected, which could provide sufficient substrate for ATP formation to meet requirement for increased phosphorylation in glycolysis. Moreover, genes in phosphate transport system were upregulated (Fig. 4). Specifically, *pstS* (Synpcc7942_2444) and *sphX* (Synpcc7942_2445) encoding phosphate-binding protein and *pstA* (Synpcc7942_2442) encoding permease protein in phosphate transport system were upregulated with Log2 (FC) of 1.83, 3.60 and 1.03, respectively, and *p* value < 0.001. *sphR* (Synpcc7942_1012) with Log2 (FC) of 1.25 and *p* value < 0.001 may indicate higher expression of phosphate regulon response regulator, which regulates phosphate-binding protein [23, 24]. Upregulated phosphate transport system and phosphate-responsive response regulator suggested that more phosphate would be transported and provided to meet increased ATP demand in phosphorylation, which was consistent with increased phosphate in YQ2-gal [25]. No changes were detected in the downstream of glycolysis, where extra ATP is generated, suggesting similar amount of ATP was obtained in YQ2-gal and WT [26]. Even though glucose was utilized in YQ2-gal, redundant ATP was not generated due to increased phosphorylation in glycolysis.

Pentose phosphate pathway is another major pathway for carbon metabolism [27], while most genes and metabolites involved in pentose phosphate pathway were not regulated in YQ2-gal compared with WT (Fig. 4). More upregulated metabolites in glycolysis and less changes in pentose phosphate pathway suggested that glucose was mainly degraded via glycolysis instead of pentose phosphate pathway in YQ2-gal.

TCA cycle as an important aerobic pathway for oxidation of carbohydrates starts with metabolite derived from glycolysis and pyruvate oxidation [28]. In YQ2-gal, decreased citrate (CTA) and isocitrate (ICT) and increased 2-ketoglutarate (KET) were detected (Fig. 4). Glucose utilization might promote reaction from CTA and ICT to KET, thus accumulating KET in YQ2-gal.

### Xylose-responsive pentose phosphate pathway and photosynthesis in engineered *S. elongatus* during photomixotrophic growth

**Fig. 5:**
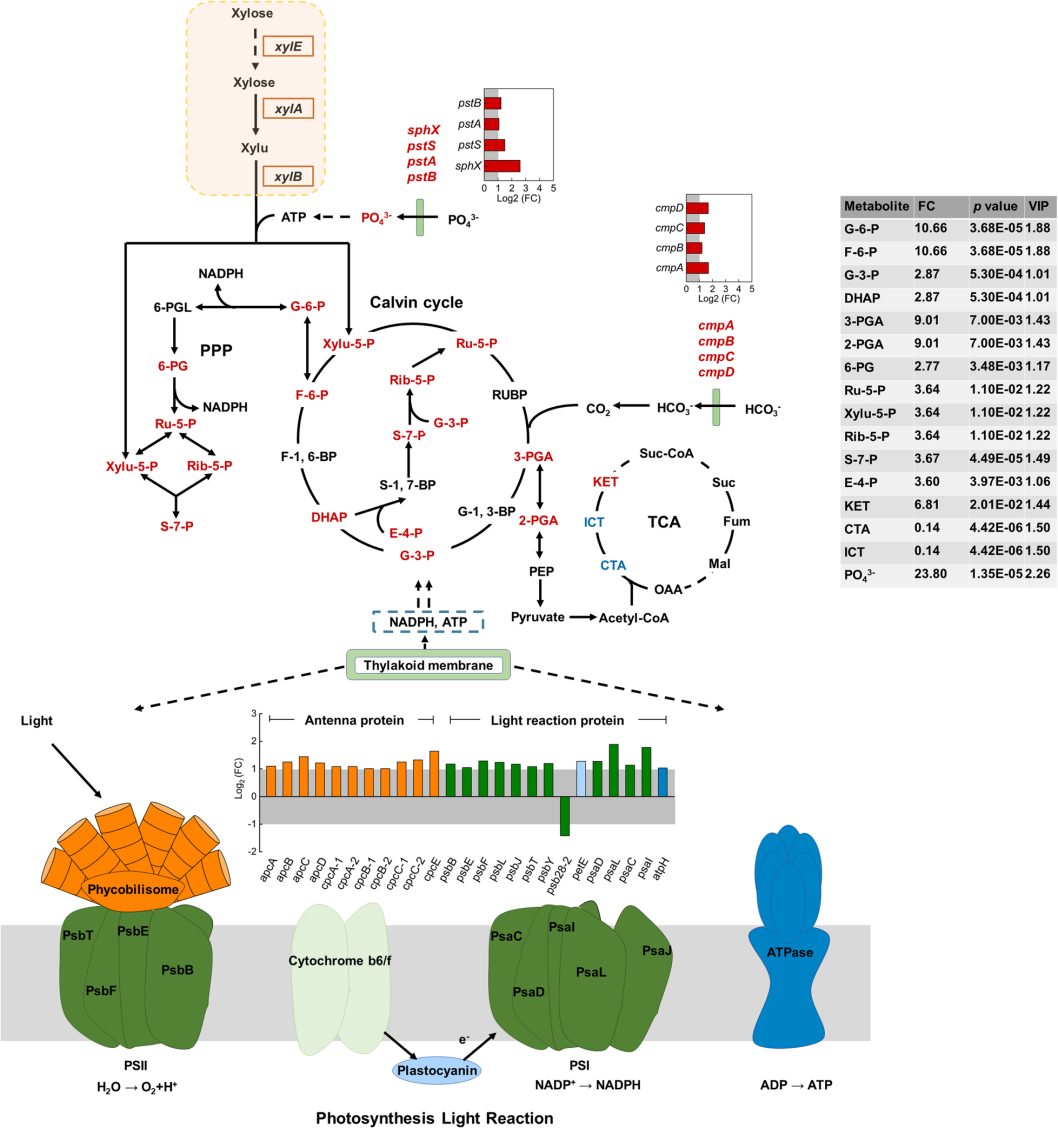
Overview of DEGs and metabolite abundance in PPP, TCA cycle and photosynthesis in YQ3-xyl compared with WT. DEGs were identified with Log2 of fold change (FC) ≥ 1 or ≤ − 1 and *p* < 0.001 and metabolites driving difference were identified by reaching variable importance for the projection (VIP) ≥ 1, FC ≥ 1.2 or ≤ 0.833 and *p* < 0.05. Red color represents highly upregulated metabolites or genes and blue color represents highly downregulated metabolites or genes. In photosynthesis light reaction pattern, DEGs columns with different colors indicated genes involved in different reaction zones, phycobilisome (orange), PSII and PSI (green), cytochrome b6/f (light green), plastocyanin (light blue), ATPase (blue). RUBP, ribulose 1,5-bisphosphate; S-1,7-BP, sedoheptulose 1,7-bisphosphate; *gap3*, glyceraldehyde 3-phosphate dehydrogenase; PSI, photosystem I; PSII, photosystem II

Metabolites obtained from xylose degradation are important precursors in pentose phosphate pathway. When xylose was efficiently utilized in YQ3-xyl, metabolites in pentose phosphate pathway, such as xylulose-5-phosphate (Xylu-5-P), were increased with FC of 3.64 and *p* value < 0.05 (Fig. 5). Other carbon metabolism, such as TCA cycle, showed similar results as that in YQ2-gal when compared with WT. Efficient utilization of xylose decreased CTA and ICT abundance and accumulated more KET in YQ3-xyl (Fig. 5). Genes, especially those correlated with regulated metabolites, were not differentially expressed, while genes in phosphate transport system were significantly upregulated with *p* value < 0.001. Specifically, genes *pstS*, *sphX*, *pstA* and *pstB* (Synpcc7942_2441) encoding ATP-binding protein were upregulated in YQ3-xyl with Log2 (FC) of 1.49, 2.60, 1.07 and 1.21, respectively (Fig. 5 and Additional file 3). More phosphate could be transported via the improved phosphate transport system, thus providing substrates for ATP formation to generate more phosphorylated metabolites. This is in consistence with increased phosphate (with a FC of 23.80 and *p* value < 0.05) and phosphorylated metabolites (e.g., ribulose-5-phosphate and ribose-5-phosphate) in pentose phosphate pathway detected in YQ3-xyl via metabolome data (Fig. 5). Moreover, these upregulated metabolites in pentose phosphate pathway are also substrates for Calvin cycle in photosynthesis, where energy is needed to drive the reaction [27, 29]. Photosynthesis light reaction is a major process for energy generation in the wild-type *S. elongatus* [11], while pentose phosphate pathway is also an energy source [27]. Regulated pentose phosphate pathway may affect the photosynthesis in YQ3-xyl.

When exploring the expression level of genes in photosynthesis light reaction, most genes were significantly upregulated in YQ3-xyl (Fig. 5). Specifically, 11 out of 16 genes encoding antenna proteins for efficient harvesting of light showed upregulation (Fig. 5). Most genes in photosystem II (PSII), plastocyanin, photosystem I (PSI) and ATPase, where light is converted into NADPH and ATP, were significantly upregulated with Log2 (FC) ≥ 1 and *p* value < 0.001 as well (Fig. 5). Chlorophyll *a*, carotenoid and their ratio (carotenoid/chlorophyll *a*) are sensitive indicators of photosynthetic activity [30]. Specifically, chlorophyll *a* serving as major roles in both light harvesting and energy conversion showed decreased yield in YQ3-xyl compared with WT (Additional file 1: Fig. S4A) [31], indicating the reduced dependency on light for energy metabolism while extra energy source, such as pentose phosphate pathway, was provided due to xylose utilization in YQ3-xyl [32]. The yield of carotenoid had no significant change, however, carotenoid/chlorophyll *a*, which relates to the deployment of photoprotective mechanism [30, 33], were increased in YQ3-xyl (Additional file 1: Fig. S4B). Previous research has reported that energy imbalance in photosynthetic apparatus in cyanobacteria could be alleviated by photoprotective mechanisms, such as heterologous ATP-consuming pathway, to assist photosynthetic performance [34, 35]. In YQ3-xyl, even though genes in photosynthesis light reaction were significantly upregulated, energy balance could be maintained by photoprotective mechanism from ATP-consuming xylose degradation, which could be confirmed by the increase of carotenoid/chlorophyll *a*, and improved photosynthesis could be demonstrated by increased abundances of most metabolites in Calvin cycle (Fig. 5).

CO_2_ is a critical participant in Calvin cycle in photosynthesis and could be obtained from bicarbonate (HCO_3_^−^) catalyzed by carbonic anhydrase. In YQ3-xyl, *ccaA*, *ecaA* and *ccmM* encoding carbonic anhydrase did not show particular changes. Genes *cmpABCD* (Synpcc7942_1488-1491) encoding proteins in bicarbonate transport were significantly upregulated with Log2 (FC) of 1.69, 1.20, 1.40 and 1.67, respectively, and *p* value < 0.001 (Fig. 5) [36, 37]. More bicarbonate may be transported into the cell by improved bicarbonate transport system with upregulated genes, thus providing more substrates for Calvin cycle in photosynthesis.

### Regulated metabolisms of sucrose, glucosamine, fatty acids and amino acids in engineered *S. elongatus* in response to diverse sugar utilization

**Fig. 6:**
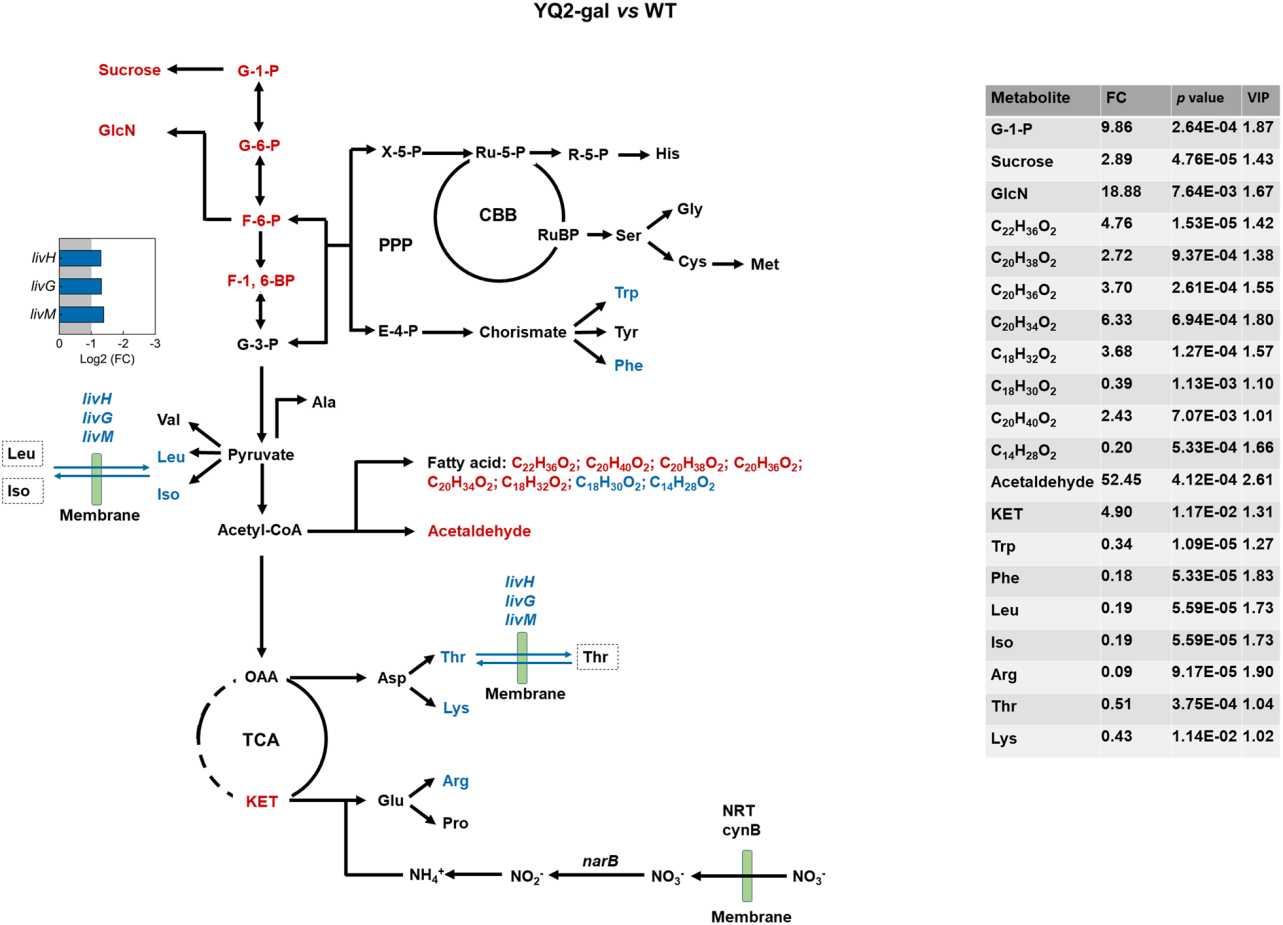
Overview of metabolite abundance and DEGs in sucrose, glucosamine, fatty acids, amino acid biosynthesis and transport in YQ2-gal compared with WT. Metabolites driving difference were identified by reaching variable importance for the projection (VIP) ≥ 1, FC ≥ 1.2 or ≤ 0.833 and *p* < 0.05. Red color represents highly upregulated metabolites or genes and blue color represents highly downregulated metabolites or genes. GlcN, glucosamine; His, Histidine; Ser, Serine; Gly, Glycine; Cys, Cysteine; Met, Methionine; Trp, Tryptophan; Tyr, Tyrosine; Phe, Phenylalanine; Ala, Alanine; Val, Valine; Leu, Leucine; Iso, Isoleucine; Asp, Aspartate; Thr, Threonine; Lys, Lysine; Glu, Glutamate; Arg, Arginine; Pro, Proline

Metabolites from glycolysis, pentose phosphate pathway, TCA cycle and Calvin cycle are important precursors for sucrose, glucosamine, fatty acids and amino acids. With utilization of glucose or xylose, regulated precursors may affect biosynthesis of these metabolites. For instance, increased G-1-P and F-6-P by efficient utilization of glucose may be a main reason for a 1.89-fold and 17.88-fold increase of sucrose and glucosamine (GlcN) in YQ2-gal when compared with that in WT (Fig. 6). Moreover, significant increase of GlcN with *p* value < 0.05 indicated that more carbon flowed into GlcN biosynthesis in YQ2-gal during photomixotrophic growth with glucose. Fatty acids are produced from acetyl-CoA [3]. Most fatty acids, especially long-chain fatty acids with more than 20 carbons, were increased in YQ2-gal (Fig. 6). Another metabolite of acetyl-CoA, acetaldehyde, was also increased with a fold change of 52.45 (*p* value < 0.05) when compared with WT (Fig. 6). Even though significant change was not detected in acetyl-CoA, increased fatty acids and acetaldehyde indicated the increased carbon flux through acetyl-CoA. With more carbon flowed into sugar and fatty acids biosynthesis, amino acids biosynthesis may be affected. In YQ2-gal, seven out of 15 amino acids had decreased abundance when compared with that in WT (Fig. 6). Moreover, genes in branched-chain amino acid transport were downregulated in YQ2-gal (Fig. 6). Genes *natD* (Synpcc7942_2495) and *livM* (Synpcc7942_2494) encoding permease and *natA* (Synpcc7942_2493) encoding ATP-binding protein in amino acid transport showed downregulation with Log2 (FC) of − 1.31, − 1.39 and − 1.31, respectively, and *p* value < 0.001 in YQ2-gal compared with WT. Amino acid transport is bidirectional and could be regulated by corresponding genes [38]. Downregulated genes would mitigate branched-chain amino acid transport, thus maintaining intracellular amino acid level (leucine, isoleucine and threonine) in YQ2-gal.

**Fig. 7:**
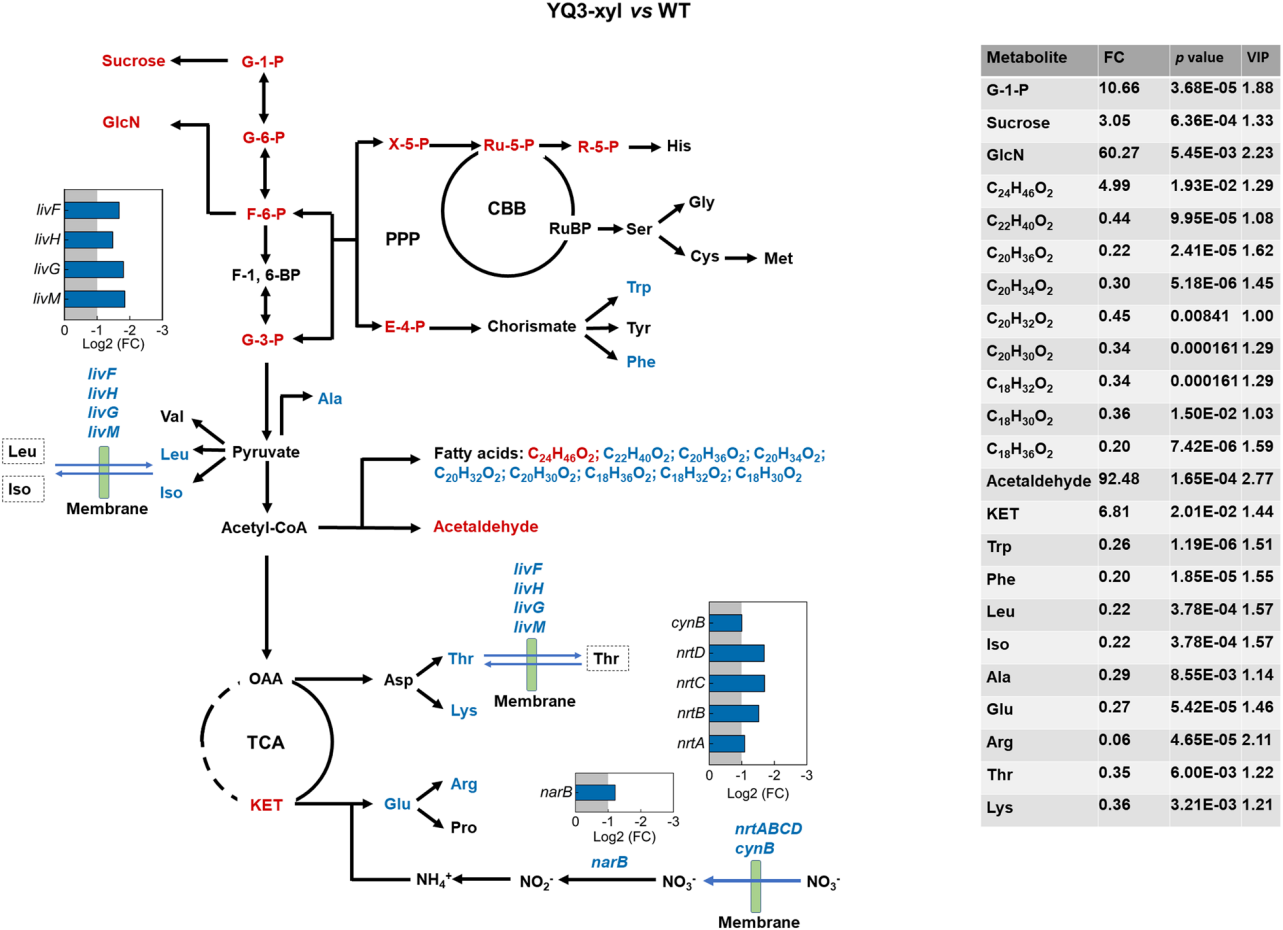
Overview of metabolite abundance and DEGs in sucrose, glucosamine, fatty acids, amino acid biosynthesis and transport in YQ3-xyl compared with WT. Metabolites driving difference were identified by reaching variable importance for the projection (VIP) ≥ 1, FC ≥ 1.2 or ≤ 0.833 and *p* < 0.05. Red color represents highly upregulated metabolites or genes and blue color represents highly downregulated metabolites or genes

Similar results of increased sucrose with a FC of 3.05 (*p* value < 0.05) and GlcN with a FC of 60.27 (*p* value < 0.05) were also detected in YQ3-xyl (Fig. 7). However, most fatty acids were decreased in YQ3-xyl (Fig. 7). More sugars and acetaldehyde (92.48-fold with *p* value < 0.05) production indicated that carbon was redistributed with less into fatty acids biosynthesis in YQ3-xyl. In amino acids metabolism, decreased amino acid and downregulated gene expression in amino acid transport were also detected in YQ3-xyl when compared with WT (Fig. 7). In addition, genes in nitrate (NO_3_^−^) transport and assimilation were downregulated. In *Synechococcus*, extracellular nitrate is first transferred into cells by passive diffusion or by active transport mediated via ABC-type transporters encoded by *nrtA*(Synpcc7942_1239), *nrtB* (Synpcc7942_1238), *nrtC* (Synpcc7942_1237) and *nrtD* (Synpcc7942_1236), then nitrate could be reduced to nitrite (NO_2_^−^) catalyzed by nitrate reductase encoded by *narB* (Synpcc7942_1235) and further reduced to ammonia (NH_4_^+^), which is a major substrate for amino acid biosynthesis, especially for glutamate biosynthesis. In YQ3-xyl, *nrtABCD* were downregulated with Log2 (FC) ≤ − 1 and *p* value < 0.001 (Fig. 7). Gene *cynB* (Synpcc7942_2106) encoding ABC-type cyanate transporter which could transport nitrate, showed downregulation as well [39]. *narB* in YQ3-xyl was negatively expressed with Log2 (FC) of − 1.21 and *p* < 0.001. Downregulation of genes in nitrate transport and assimilation to nitrite may affect ammonia production, thus impacting on glutamate biosynthesis (Fig. 7). This is consistent with decreased abundance of glutamate in YQ3-xyl.

### Perspectives for photomixotrophic bioproduction in cyanobacteria

**Fig. 8:**
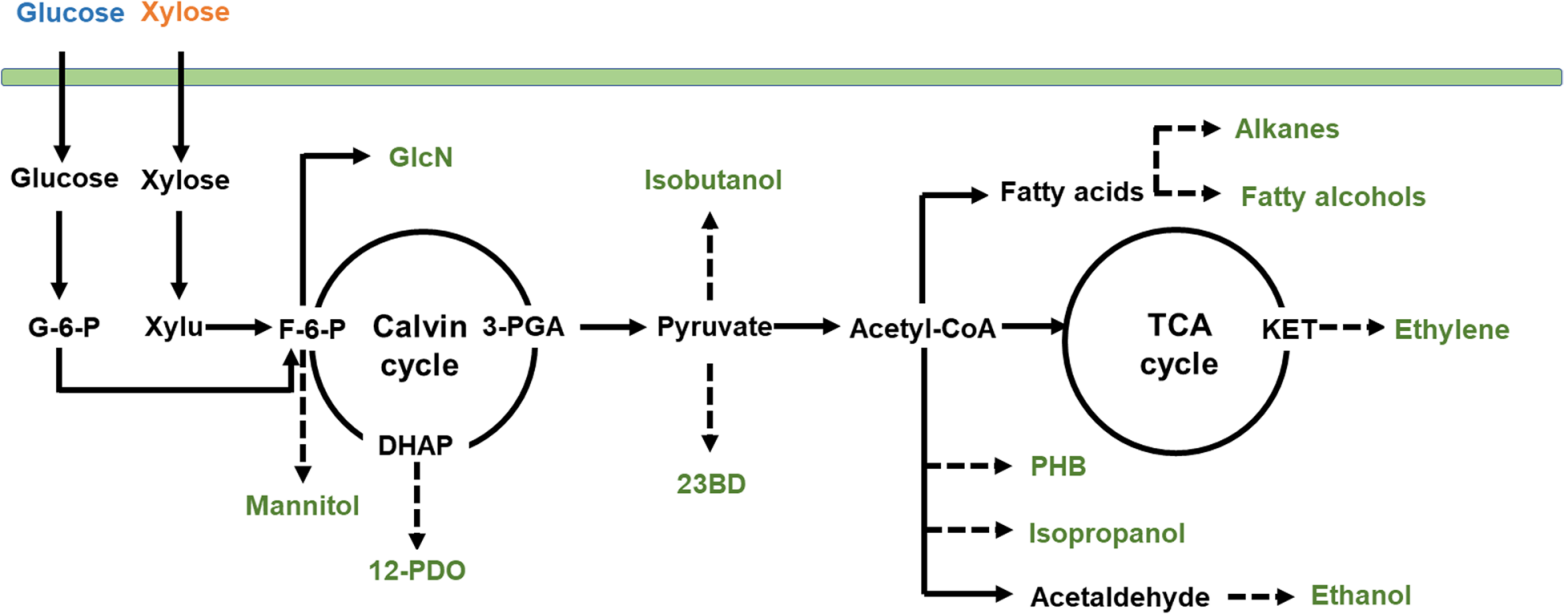
Chemical production from cyanobacteria. Chemicals in green are produced from cyanobacteria. Dashed arrow indicated that introductions of heterologous pathways are necessary. 23BD, 2,3-butanediol; PHB, poly-β-hydroxybutyrate; 12-PDO, 1, 2-propanediol

Cyanobacteria have been engineered to produce a wide range of chemicals, and synthetic photomixotrophic strains have been applied as chassis to further increase the productivity using additional carbon sources (Fig. 8) [13, 40]. For instance, the productivity of 23BD and isobutanol could be significantly increased in cyanobacteria with utilization of glucose or xylose [14, 17]. In the presence of xylose, an ethylene-producing strain with introduction of xylose degradation pathway enhanced the ethylene production [10], partially due to the increased substrate of KET.

Even though glucose or xylose has not been applied in other chemical production, increased F-6-P, DHAP and acetaldehyde indicated that production of mannitol, 1, 2-propanediol (12-PDO) and ethanol has great potentials to be increased in cyanobacteria with efficient utilization of sugars, since sufficient precursors are provided (Fig. 8) [41–44]. Other substrates (e.g., acetyl-CoA) for chemical production (e.g., isopropanol and poly-β-hydroxybutyrate, PHB) were not detected with regulations by sugar utilization (Fig. 8) [45, 46]. However, regulated metabolites (e.g., acetaldehyde) indicated the changed carbon flux through the precursors. To increase the productivity of desired chemicals, modifications of synthetic pathway, such as overexpressing related genes, and repressing genes in bypass, may be an efficient way to utilize sugars during bioproduction. Moreover, sucrose functions both as osmoprotectant and as an energy reserve in cyanobacteria [47, 48]. Increased sucrose accumulation in cyanobacteria with efficient utilization of both sugars suggested the enhanced tolerance to stress conditions and the promoted metabolism under dark condition. GlcN as a dietary supplement for joint health also showed significant increase with both sugar utilization [49], suggesting a potential drug production in synthetic photomixotrophic cyanobacteria.

However, metabolites, such as fatty acids, showed dramatically different changes in synthetic photomixotrophic cyanobacteria when compared with WT. Most fatty acids were increased in glucose-consuming strain, while decreased in xylose-consuming strain. Therefore, effect of different sugars should be considered and optimization of synthetic pathways should be conducted during photomixotrophic production of fatty alcohols and alkanes (Fig. 8) [50, 51]. Detailed information from transcriptomic and metabolic analysis of cyanobacteria during photomixotrophic growth could bring up new insights and offer valuable guidance to photomixotrophic bioproduction using this potential chassis.

## Conclusions

Due to the redirected carbon flux and transcriptional perturbations, carbon metabolism, fatty acids and amino acid synthesis, and membrane transport were regulated in engineered *S. elongatus* during photomixotrophic growth with sugars, where fatty acids metabolism was differently regulated in response to glucose and xylose utilization. Moreover, regulated photosynthesis in engineered *S. elongatus* with xylose utilization indicated that light dependency could be modulated under photomixotrophic condition. Since these metabolic regulations are closely correlated with chemical productions, we envision this study would bring some guidance for photomixotrophic bioproduction using this rising microbial chassis.

## Methods

### Chemicals and reagents

All the chemicals used in this study were at analytical grade and purchased from the Sinopharm Chemical Reagent company (China) unless otherwise specified. Enzymes and kits used for molecular cloning were purchased from New England Biolabs (NEB, USA). Oligonucleotides were synthesized by Beijing Genomics Institute (BGI, China). Spectinomycin and IPTG (Isopropyl-β-d-thiogalactopyranoside) used in this study were obtained from MDBio (MDBio, Inc., China) and Biotopped (Biotopped Life Science, China), respectively.

### Strain, medium and growth conditions

All cyanobacterial strains used in this study were summarized in Table 1 and cultured in BG-11 media at 30 °C with continuous illumination of 2000–3000 lx [15]. Spectinomycin (20 μg/mL) and IPTG (0.1 mM) were added to select recombinant strains and induce the *P*_*trc*_ promoter. Glucose 5 g/L (27.78 mM) or xylose of 5 g/L (33.33 mM) was supplemented as organic carbon source.

**Table 1:** Cyanobacterial strains used in this study

Names	Description	Sources
WT	*S. elongatus* PCC7942 (wild type)	ATCC 33912
YQ1-ctrl	WT, P_Trc_-*MCS* integrated in NSI	This study
YQ2-gal	WT, P_Trc_-*galP* integrated in NSI	This study
YQ3-xyl	WT, P_Trc_-*xylEAB* integrated in NSI	This study

All *Escherichia coli* strains were cultivated in LB medium (5 g/L of yeast extract, 10 g/L of tryptone, 10 g/L of NaCl, pH 7.0) at 37 °C. *E. coli* MG1655 genomic DNA was used as a PCR template for target gene amplification. *E. coli* DH5α was used for plasmid construction and propagation. Spectinomycin (20 μg/mL) was added for selecting transformants harboring a plasmid.

### DNA manipulation, plasmid and strain construction

All primers and plasmids used in this study were listed in Additional file 1: Table S1 and S2. Genes *galP*, *xylE*, *xylA* and *xylB* were amplified from *E. coli* MG1655 genomic DNA. *galP* gene was amplified using primers G1 and G2, digested with MfeI and BgIII, and then ligated with pAM2991 digested with EcoRI and BamHI to create pAM2991-*galP*. *xylE* gene was amplified by primers X1 and X2. *xylAB* genes was amplified by primers X3 and X4. Two fragments of pAM2991 template were amplified by primers V1 and V2, V3 and V4, respectively. Gibson Assembly was applied to fuse these four gene fragments to create pAM2991-*xylEAB*. Plasmids carrying target genes or empty backbone were then transformed into *S. elongatus* following the method described previously [52]. Briefly, *S. elongatus* at exponential phase was collected and transformation was conducted at 30 °C with gentle agitation overnight in the dark. Transformants were selected by spectinomycin and correct recombinants were confirmed by colony PCR to verify integration into cyanobacterial chromosome at NSI. Cyanobacterial strains used and constructed were listed in Table 1.

### Growth assays

Cyanobacterial strains collected from exponential phase were diluted to an OD_730_ of 0.2 in 50 mL BG-11 medium including spectinomycin and IPTG. Wild-type assays omitted addition of spectinomycin and IPTG. Glucose or xylose was added to provide exogenous carbon source. Cell growth was recorded at OD_730_ every 24 h using a spectrophotometer (JINGHUA Instruments, China). Sugar concentrations were measured by high-performance liquid chromatography (HPLC, Shimadzu LC-20AT) equipped with a refractive index detector and a Rezex ROA-Organic Acid H^+^ (8%) column (Phenomenex Inc., Torrance, CA). The column was eluted with 0.005 N of H_2_SO_4_ at a flow rate of 0.6 mL/min at 50 °C.

### Pigment quantification

Chlorophyll *a* and carotenoids were measured based on a method reported previously [53]. Briefly, chlorophyll *a* and carotenoids were extracted by re-suspending the cell pellet (1 mL cell culture, centrifuged at 15,000*g* for 7 min) in 100% methanol at 4 °C for 20 min. The extract absorbance was measured at 470 nm, 665 nm and 720 nm, and concentrations of pigments were calculated according to the equations [54, 55]:

$${\text{Chlorophyll}}\,a\,\left( {\mu {\text{g}}/{\text{mL}}} \right) = {\mkern 1mu} 12.9447\left( {{\text{A665}} - {\text{A72}}0} \right)$$

$${\text{Carotenoid }}\left( {\mu {\text{g}}/{\text{mL}}} \right){\mkern 1mu} \, = {{\left( {1000\,{\mkern 1mu} \left( {{\text{A}}470 - {\text{A}}720} \right) - 2.86{\text{ }}\left( {{\text{Chlorophyll }}a\,{\mkern 1mu} \left( {\mu {\text{g}}/{\text{mL}}} \right)} \right)} \right)} \mathord{\left/ {\vphantom {{\left( {1000\,{\mkern 1mu} \left( {{\text{A}}470 - {\text{A}}720} \right) - 2.86{\text{ }}\left( {{\text{Chlorophyll }}a\,{\mkern 1mu} \left( {\mu {\text{g}}/{\text{mL}}} \right)} \right)} \right)} {221}}} \right. \kern-\nulldelimiterspace} {221}}$$

### RNA sequencing

Cells at exponential phase with three biological replicates were collected and submitted to BGI for RNA sequencing. Total RNA was isolated and purified at BGI with quality and concentration determined by Bioanalyzer 2100 (Agilent). Library construction and RNA sequencing were performed on BGISEQ-500 platform (BGI, China) using Combinational Probe-Anchor Synthesis Sequencing Method. All raw sequencing reads were trimmed based on adaptors, reads where unknown bases reached more than 10%, and low quality. Clean reads were then obtained and mapped to genome of *S. elongatus* by HISAT [56]. Gene expression level was quantified by RSEM and normalization procedures was processed with FPKM [57, 58]. DEGseq method was used to screen differentially expressed genes with Log2 (FC) ≥ 1 or ≤ − 1 and *p* value < 0.001 [59].

### Metabolomics profiling

Strains with six biological replicates were collected at exponential phase for metabolomics profiling. Metabolites were extracted as previously described [60]. Briefly, 300 μL cold methanol was added into 100 μL sample and cells were broken using TissueLyser at 50 Hz for 4 min. After standing for 2 h in − 20 °C, all samples were centrifuged at 30,000*g*, 4 °C for 20 min. Supernatants were collected for each sample. A quality control (QC) sample was made by mixing 35 μL from each sample supernatant to estimate a mean profile representing all the analytes encountered during analysis. All supernatants as well as QC sample were subjected to metabolomics profiling by 2777C UPLC system (Water, UK) coupled with mass spectrometer Xevo G2-XS QTOF (Waters, UK) in BGI. Both positive and negative mode were operated. To evaluate the stability of LC–MS during the whole acquisition, a QC sample was acquired after every 10 samples. Raw data were imported into Progenesis QI software and then preprocessed using metaX software. Features detected in less than 50% of the QC samples or less than 20% of the experimental samples were removed, and missing values were imputed using the k-nearest neighbor (KNN) method. The QC-robust spline batch correction (QC-RSC) was used to correct signal drift and batch variation. The relative s.d. (RSD) value of metabolites in the QC samples was set at a threshold of 30%, and features with a RSD less than 30% in the QC samples were retained [60]. After normalization and filtering, univariate and multivariate were conducted by metaX. To identify metabolites, standards were used and molecular mass data were matched to KEGG and BGI own database Met-Lib for a further check. Metabolites driving differences were identified by reaching variable importance for the projection (VIP) ≥ 1, FC ≥ 1.2 or ≤ 0.833 and *p* value < 0.05.

## Supplementary Information

**Additional file 1: Fig. S1** Sugar consumption profiles of WT (empty circle), YQ1-ctrl (gray circle) and YQ2-gal (blue circle) in BG-11 medium with 5 g/L glucose (A) and WT (empty circle), YQ1-ctrl (gray circle) and YQ3-xyl (orange circle) in BG-11 medium with 5 g/L xylose (B). Error bars represent standard deviations (in duplicate). **Fig. S2** Principal component analysis (PCA) of transcriptomics data for replicates of YQ2-gal and WT (A) and YQ3-xyl and WT (B). **Fig. S3** Principal component analysis (PCA) of metabolomics data for replicates of YQ2-gal and WT (A, B), and YQ3-xyl and WT (C, D). Specifically, A and C represents the results in positive ion mode, B and D represents the results in negative ion mode. **Fig. S4** Pigment measurement (A) and carotenoid/chlorophyll *a* (B) of WT (white) during photoautotrophic growth and YQ3-xyl (orange) during photomixotrophic growth. Error bars represent standard deviations (in triplicate). * represents significant difference with *p* value less than 0.05, ** represents significant difference with *p* value less than 0.01. **Table S1** Primers used in this study. **Table S2** Plasmids used in this study.

**Additional file 2. **DEGs in YQ2-gal strain during photomixotrophic growth with glucose.

**Additional file 3. **DEGs in YQ3-xyl strain during photomixotrophic growth with xylose.

## Data Availability

RNA-seq data are available at the NCBI Sequence Read Archive (SRA) under accession number PRJNA729175. Raw metabolomics data are available on Metabolights under accession number MTBLS2825.

## Abbreviations

CO_2_: Carbon dioxide
RuBisCO: Ribulose-1,5-bisphosphate carboxylase/oxygenase
DEGs: Differentially expressed genes
FC: Fold change
NSI: Neutral site I
PPP: Pentose phosphate pathway
TCA cycle: Tricarboxylic acid cycle
Glu: Glucose
Xyl: Xylose
Xylu: Xylulose
Xylu-5-P: Xylulose-5-phosphate
G-6-P: Glucose 6-phosphate
F-6-P: Fructose 6-phosphate
F-1,6-BP: Fructose 1,6-bisphosphate
G-3-P: Glyceraldehyde 3-phosphate
DHAP: Dihydroxyacetone phosphate
G-1,3-BP: Glycerate 1,3-diphosphate
3-PGA: 3-Phosphoglycerate
2-PGA: 2-Phospho-(R)-glycerate
PEP: Phosphoenolpyruvate
CTA: Citrate
ICT: Isocitrate
KET: 2-Ketoglutarate
6-PGL: Glucono-1,5-lactone 6-phosphate
6-PG: 6-Phospho-d-gluconate
Ru-5-P: Ribulose 5-phosphate
Rib-5-P: Ribose 5-phosphate
S-7-P: Sedoheptulose 7-phosphate
E-4-P: Erythrose 4-phosphate
RUBP: Ribulose 1,5-bisphosphate
S-1,7-BP: Sedoheptulose 1,7-bisphosphate
*gap3*: Glyceraldehyde 3-phosphate dehydrogenase
PSI: Photosystem I
PSII: Photosystem II
GlcN: Glucosamine
His: Histidine
Ser: Serine
Gly: Glycine
Cys: Cysteine
Met: Methionine
Trp: Tryptophan
Tyr: Tyrosine
Phe: Phenylalanine
Ala: Alanine
Val: Valine
Leu: Leucine
Iso: Isoleucine
Asp: Aspartate
Thr: Threonine
Lys: Lysine
Glu: Glutamate
Arg: Arginine
Pro: Proline
23BD: 2,3-Butanediol
PHB: Poly-β-hydroxybutyrate
IPTG: Isopropyl-β-d-thiogalactopyranoside
VIP: Variable importance for the projection
