# Supplementary material for: Transcriptomics and metabolomics of engineered Synechococcus elongatus during photomixotrophic growth
Source: Microb Cell Fact. 2022 Mar 5;21:31. doi: 10.1186/s12934-022-01760-1 (PMC8897908; doi:10.1186/s12934-022-01760-1)
Supplement: Supplementary file 1 — Additional file 1: Fig. S1 Sugar consumption profiles of WT (empty circle), YQ1-ctrl (gray circle) and YQ2-gal (blue circle) in BG-11 medium with 5 g/L glucose (A) and WT (empty circle), YQ1-ctrl (gray circle) and YQ3-xyl (orange circle) in BG-11 medium with 5 g/L xylose (B). Error bars represent standard deviations (in duplicate). Fig. S2 Principal component analysis (PCA) of transcriptomics data for replicates of YQ2-gal and WT (A) and YQ3-xyl and WT (B). Fig. S3 Principal component analysis (PCA) of metabolomics data for replicates of YQ2-gal and WT (A, B), and YQ3-xyl and WT (C, D). Specifically, A and C represents the results in positive ion mode, B and D represents the results in negative ion mode. Fig. S4 Pigment measurement (A) and carotenoid/chlorophyll a (B) of WT (white) during photoautotrophic growth and YQ3-xyl (orange) during photomixotrophic growth. Error bars represent standard deviations (in triplicate). * represents significant difference with p value less than 0.05, ** represents significant difference with p value less than 0.01. Table S1 Primers used in this study. Table S2 Plasmids used in this study. [file 12934_2022_1760_MOESM1_ESM.docx]

**Additional file 1**

**Transcriptomics and metabolomics of engineered Synechococcus elongatus during photomixotrophic growth**

Lin-Rui Tan^1^, Yi-Qi Cao^1^, Jian-Wei Li^1^, Peng-Fei Xia^1^, Shu-Guang Wang^1, 2#^

^1^ Shandong Key Laboratory of Water Pollution Control and Resource Reuse, School of Environmental Science and Engineering, Shandong University, Qingdao 266237, China

^2^ Sino-French Research Institute for Ecology and Environment (ISFREE), Shandong University, Qingdao 266237, China

# Address corresponding to Shu-Guang Wang, wsg@sdu.edu.cn

**
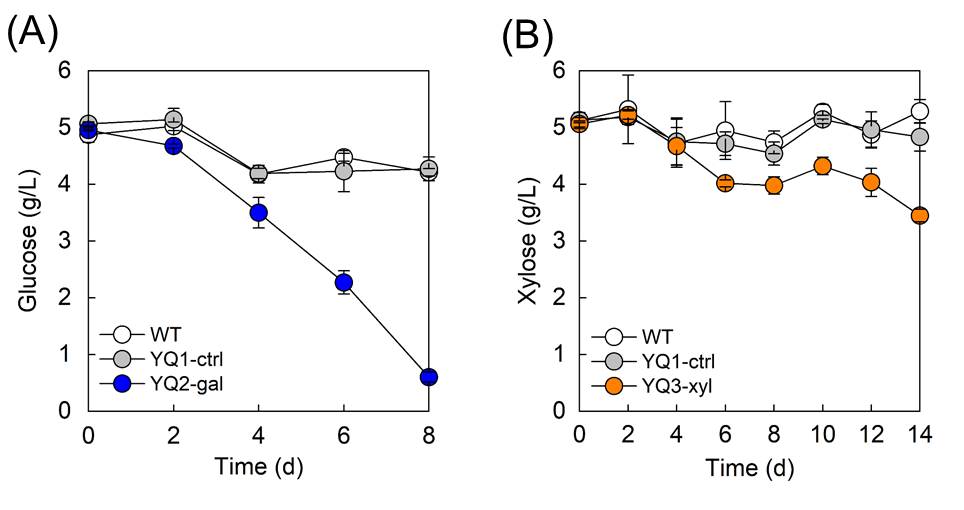
**

**Fig. S1** Sugar consumption profiles of WT (empty circle), YQ1-ctrl (gray circle) and YQ2-gal (blue circle) in BG-11 medium with 5 g/L glucose (A) and WT (empty circle), YQ1-ctrl (gray circle) and YQ3-xyl (orange circle) in BG-11 medium with 5 g/L xylose (B). Error bars represent standard deviations (in duplicate).


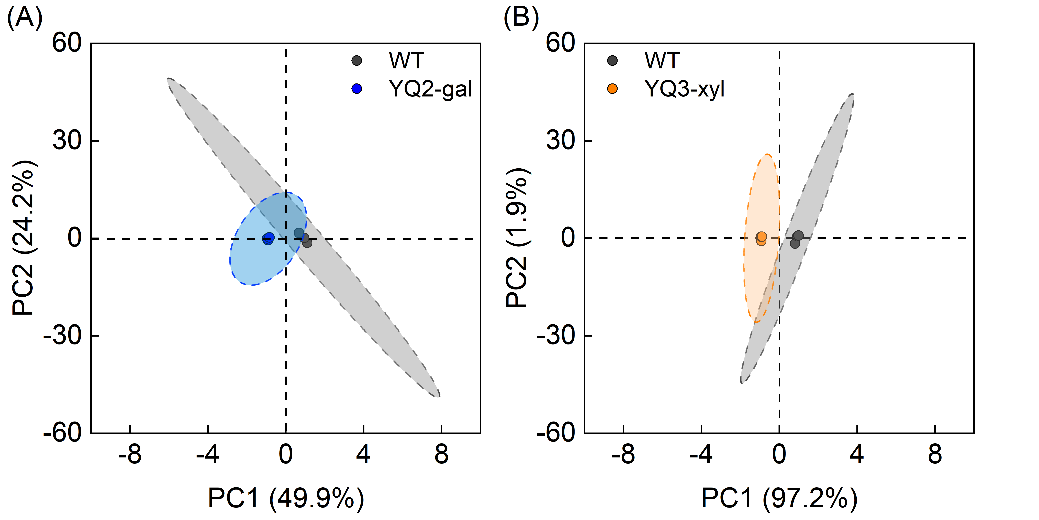


**Fig. S2** Principal component analysis (PCA) of transcriptomics data for replicates of YQ2-gal and WT (A) and YQ3-xyl and WT (B).


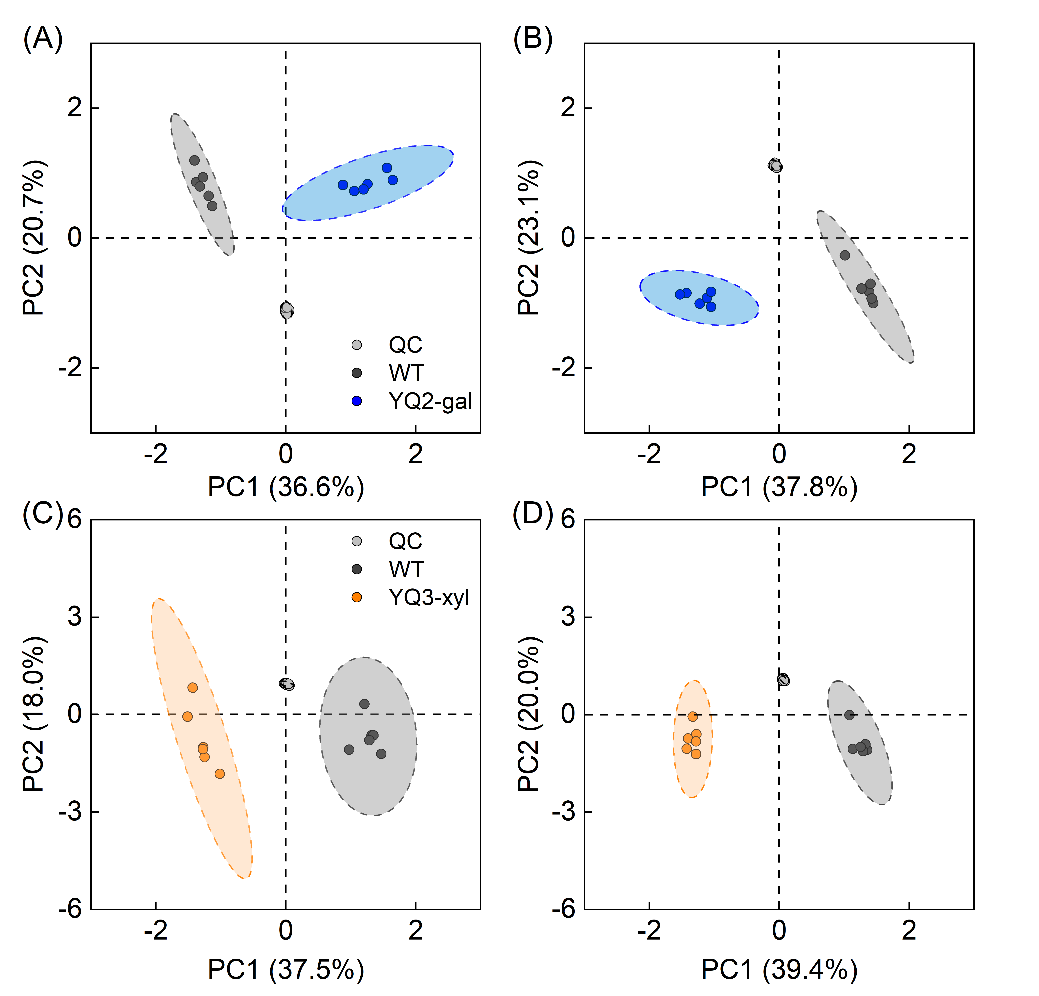


**Fig. S3** Principal component analysis (PCA) of metabolomics data for replicates of YQ2-gal, WT and QC (quality control) (A, B), and YQ3-xyl, WT and QC (C, D). Specifically, A and C represents the results in positive ion mode, B and D represents the results in negative ion mode.

**
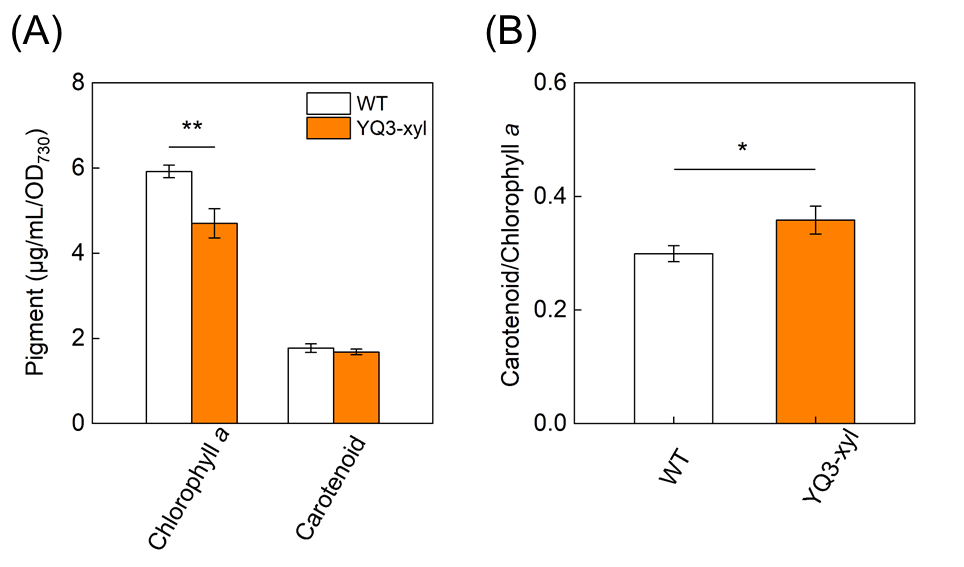
**

**Fig. S4** Pigment measurement (A) and carotenoid/chlorophyll *a* (B) of WT (white) during photoautotrophic growth and YQ3-xyl (orange) during photomixotrophic growth. Error bars represent standard deviations (in triplicate). * represents significant difference with *p* value less than 0.05, ** represents significant difference with *p* value less than 0.01.

**Table S1 Primers used in this study.**

| Primer names | Sequences |
| --- | --- |
| ***Gene cloning*** |  |
| G1 | ctaacaattgatgcctgacgctaaaaaacaggggcg |
| G2 | ctatagatctttaatcgtgagcgcctatttcgcgcagtt |
| X1 | atttcacacaggaaacagaccatggaattcaatacccagtataattccagttatatattttcga |
| X2 | gcgatcgagctggtcaaaataggcttgcatttacagcgtagcagtttgttgtgttttct |
| X3 | agaaaacacaacaaactgctacgctgtaaatgcaagcctattttgaccagctcgatcgc |
| X4 | cttctgcgttctgatttaatctgtatcaggttacgccattaatggcagaagttgctgatagagg |
| V1 | atcagcaacttctgccattaatggcgtaacctgatacagattaaatcagaacgcagaagcgg |
| V2 | tgattctgtggataaccgtattaccgcctttgagtg |
| V3 | cactcaaaggcggtaatacggttatccacagaatca |
| V4 | tcgaaaatatataactggaattatactgggtattgaattccatggtctgtttcctgtgtgaaat |
| ***Gene confirmation*** |  |
| Conf-G1 | tttttcgtctgcttccttgc |
| Conf-G2 | tgtttggtttccggtacca |
| Conf-G3 | tttggcctggatatcggtgt |
| Conf-G4 | gatgaactcgacgaggccat |
| Conf-X1 | gtatcctgcgcaaaattatg |
| Conf-X2 | gcattctcttccacactg |
| Conf-X3 | gggtaagcgtatggaagagc |
| Conf-X4 | ttcagctgctttaggccc |

Restriction sites are underlined.

**Table S2 Plasmids used in this study.**

| Names | Description | Reference |
| --- | --- | --- |
| pAM2991 | NSI targeting vector; *P_trc_*-*MCS* | Addgene #40248 |
| pAM2991-*galP* | pAM2991, *P_trc_*-*galP* | This study |
| pAM2991-*xylEAB* | pAM2991, *P_trc_*-*xylEAB* | This study |
